# Supplementary material for: Genome-wide CRISPR Screens in T Helper Cells Reveal Pervasive Crosstalk between Activation and Differentiation
Source: Cell. 2019 Feb 7;176(4):882–896.e18. doi: 10.1016/j.cell.2018.11.044 (PMC6370901; doi:10.1016/j.cell.2018.11.044)
Supplement: Data S2. Processed Data from All the Steps of the Analysis, Related to Figure 1 [file mmc2.zip › supplemental data/motif analysis/Pparg_homer/homerResults/motif15.similar.html]

motif15

## Information for motif15

A
G
T
C
G
T
A
C
A
G
T
C
G
A
T
C
A
G
T
C
G
T
A
C
G
T
A
C
G
T
A
C
G
T
A
C
G
T
A
C
  
Reverse Opposite:  

C
A
T
G
C
A
T
G
C
A
T
G
C
A
T
G
C
A
T
G
T
C
A
G
C
T
A
G
T
C
A
G
C
A
T
G
T
C
A
G
  

|  |  |
| --- | --- |
| p-value: | 1e-40 |
| log p-value: | -9.356e+01 |
| Information Content per bp: | 1.644 |
| Number of Target Sequences with motif | 1661.0 |
| Percentage of Target Sequences with motif | 13.27% |
| Number of Background Sequences with motif | 3423.2 |
| Percentage of Background Sequences with motif | 9.55% |
| Average Position of motif in Targets | 101.6 +/- 58.1bp |
| Average Position of motif in Background | 102.3 +/- 55.5bp |
| Strand Bias (log2 ratio + to - strand density) | -0.0 |
| Multiplicity (# of sites on avg that occur together) | 1.16 |
| Motif File: | file (matrix) reverse opposite |

### Similar de novo motifs found

|  |  |  |  |  |  |  |  |
| --- | --- | --- | --- | --- | --- | --- | --- |
| Rank | Match Score | Redundant Motif | P-value | log P-value | % of Targets | % of Background | Motif file |
| 1 | 0.858 | T C A G A C T G A C T G A T C G T A C G C T A G C T A G T C A G | 1e-35 | -82.098281 | 10.94% | 7.77% | motif file (matrix) |
| 2 | 0.968 | A T C G C T A G A C T G A T C G A T C G A C T G A T C G A T C G C T A G A T C G A T C G T C A G | 1e-34 | -80.503682 | 14.99% | 11.32% | motif file (matrix) |
| 3 | 0.629 | A G T C G A C T A C T G T A G C G A T C A G T C A T G C C T A G A T G C A G T C A G C T G A T C | 1e-10 | -24.734039 | 0.42% | 0.14% | motif file (matrix) |
| 4 | 0.697 | C G A T A C T G T C A G C T A G C A T G C A T G C G A T A C T G C A T G A C T G C T A G A T C G | 1e-5 | -13.667383 | 0.91% | 0.56% | motif file (matrix) |
